# Pandora's Box

**DOI:** 10.1192/bji.2024.7

**Published:** 2024-05

## Down with the dictatorship of papers

The methods used to evaluate scientific work have evolved over time, but volume of publications has continued to tyrannise scientists and has remained a key criterion for career progress. Readers may have noticed the large expansion of journals pursuing scientists and offering rapid processing of papers.

In an attempt to increase research productivity, in 1994, the Spanish government introduced a system of assessment based on volume of publications. Although at face value this succeeded, and by 2021 Spain ranked 11th in terms of global scientific output, academics criticised the system as having a negative impact on quality of work. Researchers feeling the pressure started putting quantity before quality, cutting corners and producing low-quality papers; some were accused of receiving payments to fraudulently co-author papers in which they had no involvement. It was also observed that academics were encouraged to prioritise research over teaching and that public funds were being used to pay the increasing processing fees imposed by journals.

Spain has finally decided to grasp the nettle and has prepared new proposals for its National Evaluation and Accreditation Agency. Assessments under the new system take into consideration a broader range of research outputs, which include – in addition to publications – patents, reports and other outputs relevant to the area of research work. The evaluation will take into account, in addition to the impact factors of the journals in which scientists publish, whether the research reaches non-academic audiences in news reports and government documents. More value will be attached to papers that are co-produced with non-academic authors and local communities. The assessment will take into consideration the use of public funds spent on publication costs and publishing on non-commercial, open-access platforms that do not incur publishing fees.

The new system was put in place after consultation in January 2024. Time will show its success or lack of.

de los Ángeles Orfila M. Spain wants to change how it evaluates scientists – and end the ‘dictatorship of papers’. *ScienceInsider*, 29 Nov 2023.

## Suicide or euthanasia?

Euthanasia remains a major issue that divides public opinion and presents a serious ethical matter for doctors and the law. Although legalised in some European countries, Canada and New Zealand, it remains illegal in most countries, including the UK. The law as it applies at present in England and Wales, under the Suicide Act 1961, states that anyone who ‘aids, abets, counsels or procures the suicide of another’ is liable to up to 14 years in prison. In Scotland, there is no specific crime of assisting a suicide, but helping a person to die could lead to prosecution for culpable homicide. Amendments to the UK law have been periodically proposed, but no change has been made so far. A major consideration for many is the process of implementation of any law allowing euthanasia and how to safeguard vulnerable individuals. Studies examining the implementation of assisted dying or euthanasia in countries where it is legalised have raised serious questions and have been discussed by Pandora in the past (ref).

Data from the Office of National Statistics on suicide in April 2022 showed that people with severe progressive conditions such as low-survival cancer, ischaemic heart disease and chronic obstructive airway disease are twice as likely to commit suicide compared with the general population. Following these findings, the co-chair of the All-Party Parliamentary Group for Choice at the End of Life stated: ‘That so many terminally ill people feel their only option is to take their own life is a damning indictment on end-of-life choice in this country, and entirely unacceptable in a healthcare system that supposedly values patient safety and compassion […] It is now incumbent that we act on this new evidence, which indicates serious failings in the protection of our terminally ill citizens and which demands that we investigate in full the deeply concerning impact of the current law’.

But where do we, doctors, stand? After all, we are the ones who are being asked to provide the means of ending life. A BMA survey of 30 000 doctors in 2020 found that half of those that took part supported a change in the law on prescribing drugs for eligible patients to self-administer to end their life. However, when asked about administering the lethal drugs, 37% were in favour, with 47% against. A letter from 1700 doctors to the Health Secretary warned that any change in the law on physician-assisted dying would ‘threaten society's ability to safeguard vulnerable patients from abuse, it would undermine the trust the public places in physicians, and it would send a clear message to our frail, elderly and disabled patients about the value that society places on them as people’.

The pressure from various groups to consider euthanasia is not going to stop, and we doctors need to decide where we draw the line. Where do we as psychiatrists stand on euthanasia? Readers’ comments and views are welcome.

*BMA Survey on Physician-Assisted Dying*. Research Report. Kantar, 2020. Available from: https://www.bma.org.uk/media/3367/bma-physician-assisted-dying-survey-report-oct-2020.pdf.

Care Not Killing. *What We Do*. Available from: www.carenotkilling.org.uk/about/what-we-do.

Euthanasia for people with a mental illness? *BJPsych International*. 2016;**13**(2):50-51. doi:10.1192/S2056474000001197.

## Brain talk

Have you ever wondered how our brain produces speech? A recent study examined this process, with fascinating results. As the senior author, a neurosurgeon at Harvard Medical School, says, ‘Our brains perform these feats surprisingly fast – about three words per second in natural speech – with remarkably few errors. Yet how we precisely achieve this feat has remained a mystery’.

Using ultra-high-density Neuropixels probes, the researchers were able to record neuronal activity in the language-dominant prefrontal cortex, which encodes phonetic, syllabic and morphological components of planned words. They carried out single-neuron recordings in the language-dominant prefrontal cortex in participants who were undergoing planned intraoperative neurophysiology at the time. These recordings were performed in the posterior middle frontal gyrus, which is known to be involved in word planning and construction of sentences and to connect with the neighbouring motor frontal area involved in articulation. Although it did not comprehensively test all relevant processes involved in language production, this study identified specific neurons dedicated to language. It also demonstrated the value of high-density recordings in examining neuronal function. The authors speculate that their findings and further progress in this area of research could not only enable better understanding of language and speech production but also lead to the development of treatments for language disorders.

Khanna AR, Muñoz W, Kim YJ, Kfir Y, Paulk AC, Jamali M, et al Single-neuronal elements of speech production in humans. Nature 2004; 626: 603–10.10.1038/s41586-023-06982-wPMC1086669738297120

## Of men and women's work experience

Over the past years, increasing numbers of women have entered the medical profession and reached senior levels. However, are women accepted by male colleagues and respected as equal? A recent survey revealed a shocking reality, at least in the surgical workforce.

An observational study in the UK, using National Health Service workforce population data, examined experiences with sexual misconduct, including sexual harassment, sexual assault and rape among colleagues, in the past 5 years. It also examined participants’ views on the adequacy of accountable organisations in dealing with this issue. Around 1700 individuals participated, 51% of whom were women. The researchers found that compared with men, women were significantly more likely to report witnessing or being a target of sexual misconduct; 63% of women reported being a target of sexual harassment, compared with 23.7% of men. About 30% of women versus 7% of men had been sexually assaulted, and 15% and 31% of women gave low evaluations of the General Medical Council and the Royal Colleges, compared with men's evaluations of 49% and 60%, respectively.

It is unlikely that this problem is limited to the surgical specialties, and similar studies in other specialties are needed.

Begeny CT, Arshad H, Cuming T, Dhariwal DK, Fisher RA, Franklin MD, et al Sexual harassment, sexual assault and rape by colleagues in the surgical workforce, and how women and men are living different realities: observational study using NHS population-derived weights. Br J Surg 2023; 110(11): 1518–26.37697690 10.1093/bjs/znad242PMC10564399

